# Site-Specific RNase A Activity Was Dramatically Reduced in Serum from Multiple Types of Cancer Patients

**DOI:** 10.1371/journal.pone.0096490

**Published:** 2014-05-07

**Authors:** Weiyan Huang, Mei Zhao, Na Wei, Xiaoxia Wang, Huqing Cao, Quan Du, Zicai Liang

**Affiliations:** 1 Institute of Molecular Medicine, Peking University, Beijing, People’s Republic of China; 2 Department of Etiology and Carcinogenesis and State Key Laboratory of Molecular Oncology, Cancer Institute and Hospital, Chinese Academy of Medical Sciences and Peking Union Medical College, Beijing, People’s Republic of China; 3 Institute of Molecular Medicine, and the State Key Laboratory of Natural and Biomimetic Drugs, School of Pharmaceutical Sciences, Peking University, Beijing, People’s Republic of China; Meharry Medical College, United States of America

## Abstract

Potent RNase activities were found in the serum of mammals but the physiological function of the RNases was never well illustrated, largely due to the caveats in methods of RNase activity measurement. None of the existing methods can distinguish between RNases with different target specificities. A systematic study was recently carried out in our lab to investigate the site-specificity of serum RNases on double-stranded RNA substrates, and found that serum RNases cleave double-stranded RNAs predominantly at 5′-U/A-3′ and 5′-C/A-3′ dinucleotide sites, in a manner closely resembling RNase A. Based on this finding, a FRET assay was developed in the current study to measure this site-specific serum RNase activity in human samples using a double stranded RNA substrate. We demonstrated that the method has a dynamic range of 10^−5 ^mg/ml- 10^−1 ^mg/ml using serial dilution of RNase A. The sera of 303 cancer patients were subjected to comparison with 128 healthy controls, and it was found that serum RNase activities visualized with this site-specific double stranded probe were found to be significantly reduced in patients with gastric cancer, liver cancer, pancreatic cancer, esophageal cancer, ovary cancer, cervical cancer, bladder cancer, kidney cancer and lung cancer, while only minor changes were found in breast and colon cancer patients. This is the first report using double stranded RNA as probe to quantify site-specific activities of RNase A in a serum. The results illustrated that RNase A might be further evaluated to determine if it can serve as a new class of biomarkers for certain cancer types.

## Introduction

RNase A is an endoribonuclease with functions in RNA metabolism and regulation of gene expression. It was found to play roles in diseases such as autoimmune diseases, renal insufficiencies and pancreas disorder [1], [2], [3], [4]. More recently, an antitumor activity was also reported for an RNase A with cytotoxic and cytostatic properties [5], [6].

In the past decades, biochemical properties of RNase A were well investigated[7]. As a unique evolutionary conserved protein family, the members of RNase A superfamily are normally composed of 124 amino acids [8]. The members of RNase A superfamily are widely expressed and present in serum and tissues of mammals[9]. Five RNases, namely RNase 1, RNase 2, RNase 3, RNase 4 and RNase 5, were identified in human serum as early as 1976 [10], [11]. RNase 1 was later found to be the human homologue of RNase A and thus is also referred to as human RNase A. RNase A is the predominant endoribonuclease component in human organs and tissues [9]. RNase 2 and RNase 3 behave with a similar ribonucleolytic function [2]. In addition to function in mRNA and 18S rRNA degradation, RNase 4 and RNase 5 were also reported to have an angiogenesis function, whereas RNase 5 in particular could promote the growth of blood vessel [1].

Ranpirnase is a RNase A superfamily member identified in frog (Rana pipiens) oocytes [12]. As the smallest member in the RNase A superfamily, ranpirnase is a single strand poly-peptide composed of 104 amino acids [12]. Currently, ranpirnase is in a clinical trial of Phase IIIb, in which, ranpirnase was tested to treat patients with unresectable malignant mesothelioma, lung cancer and leukemia, and was demonstrated to increase survival time in treated patients [13], [14]. The unexpected discovery of anti-cancer activity of ranpirnase hinted that other novel functions might be further explored for members of RNase A superfamily.

A few methods have been tested for measuring serum RNase levels. Radioactively labeled t-RNA and dsRNA substrate were used by Saxena and Ben-Artzi to determine RNase activity of angiogenin and RNase III [15], [16]. Hybridized RNA composed of a 17-mer antisense RNA strand and RNAs from T24 cell line was used in a study to characterize double-strand RNase ribonucleolytic activity[17]. In an angiogenin study, a FRET method was used by Kelemen *et al.* to investigate the catalytic efficiency of the RNase. This method, however, works only under extremely low RNase levels [18]. Surprisingly, even though these methods are similar in principle, the results obtained from these assays were not consistent. Reddi *et al.* used poly-C as the substrate to determine RNase level by measuring the remaining substrate after RNase treatment [19]. The method was used by Funakoshi and other groups in 1976 and significant relationships were obtained between RNase levels and pancreas diseases, including pancreatic cancer and pancreatitis [20], [21], [22]. In about the same time, Marabella demonstrated a method to perform RNase assay, using yeast RNA as the substrate [23]. Later on, an iodination method was used to label ribosomal RNA. Using a radioactive labeled substrate, the remaining substrate was quantitatively determined, using an auto-gamma scintillation spectrometer [24]. By means of this protocol, Kurihara *et al.* measured the RNase activity in serum, and found that serum RNase levels were not correlated with cancer histology, instead showing a correlation with some physiological index [25]. Furthermore, chemical synthesized poly-C, as well as t-RNA extracted from *E. coli*, were used as substrates in RNase assay by Kemmer *et al.* [26]. Even though measurements of serum RNase level were accomplished in these studies, insufficient capability to differentiate individual RNase in a mixture might have been the technical obstacle that prevented the researchers to reach consensus results in different serum RNase studies.

In 2003, Czauderna *et al.* investigated the serum stability of siRNA and found that the degradation was mainly resulted from endoribonuclease cleavage. They also found that, when some critical sites were modified, serum stability of the siRNAs was significantly improved [27]. Later on, Turner and Haupenthal’s studies indicated that RNase A played an essential role in serum degradation of siRNA in mammals [28], [29]. Subsequently site-specific cleavage patterns on C-A, U-G and U-A sites were identified by Volkov [30]. In a previous study performed with125 siRNAs, we further illustrated that the cleavage of double-stranded RNAs occurred mainly at two sites, 5′-C/A-3′ (5′-U/G-3′ on complementary strand) and 5′-U/A-3′ dinucleotide sites [31].

In the current study, we designed a dual fluorescently labeled RNA duplex which contained a 5′-C/A-3′ cutting site as the specific substrate for serum RNase A. We employed fluorescence resonance energy transfer (FRET) method, a highly sensitive optical technology, to study the dynamics of reaction between fluorescently labeled substrates and RNase A, to build a standard curve to correlate the RNase levels with the amount of catalytic products in order to deduce the RNase A level of the serum. Using this assay, we were able to scrutinize the RNase A activity in the serum from cancer patients. We compared the RNase A level of patients with 11 kind of cancers with that of healthy controls, and found the serum RNase A level in patients with cervical cancer, esophageal cancer, kidney cancer, lung cancer, bladder cancer, pancreatic cancer, ovary cancer, liver cancer and gastric cancer are significantly down-regulated when compared with that of healthy controls (P<0.001), while the serum RNase level of breast cancer and colon cancer patients was not very significantly different from that of healthy individuals.

## Results

### Design and Characterization of a FRET Probe for Serum RNase Measurement

A well-known fact in molecular biology is that single-stranded RNAs are very unstable in most circumstances, particularly in the presence of ribonucleases. The fast degradation processes make it too hard to monitor the degradation process in real-time. This situation further leads to the difficulties in establishing quantitative measurements of RNase activity.

**Figure 1:**
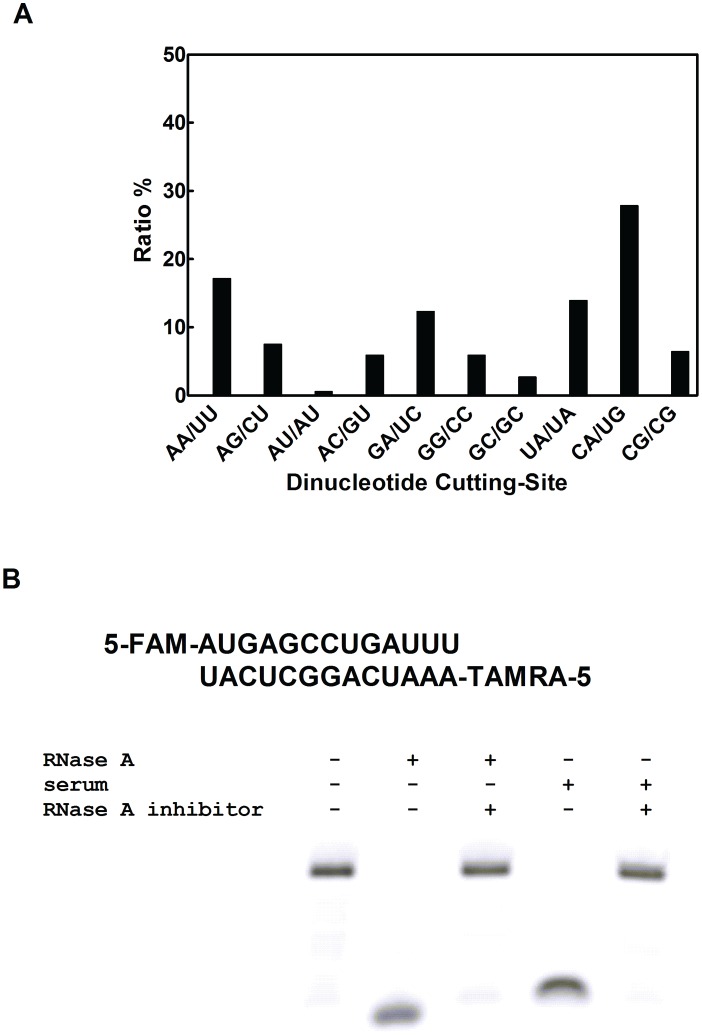
Cleavage preferences of duplex RNAs. A) Degradation of an 1860 bp long RNA duplex was performed in RNase A. Degradation fragments were extracted and sequenced. By aligning the degradation fragments with the original RNA sequence, cleavage sites were identified and presented in terms of the ratio for each possible dinucleotide site [31]. B) Duplex RNA degradation assay. Dual-labeled duplex RNA was separately incubated with RNase A, the mixture of RNase A and RNase A inhibitor, human serum, the mixture of human serum and RNase A inhibitor. Samples were collected and run in a denatured PAGE gel.

**Figure 2:**
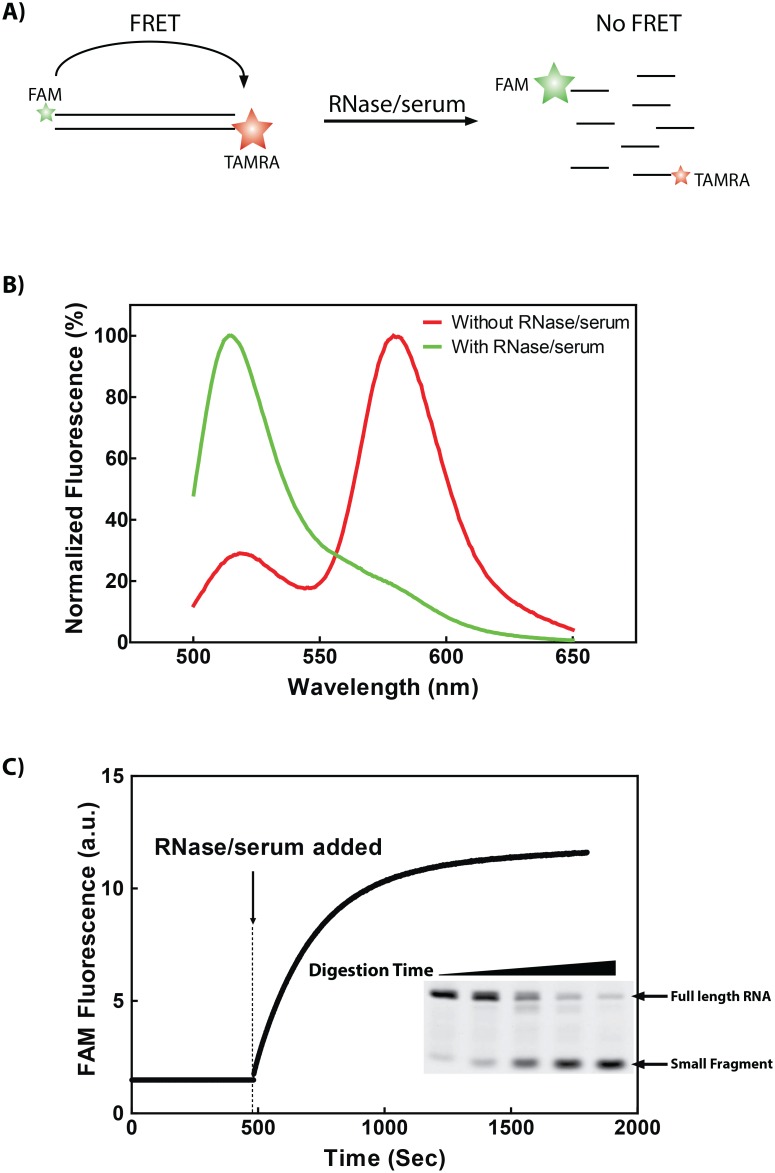
Real-time monitoring of RNase activity by fluorescence resonance energy transfer (FRET) via short double strand RNA degradation. A) Schematic illustration of the FRET based RNase assay. The duplex RNA (13 bp) was dual-labeled with a fluorescein (FAM) as donor and tetramethylrhodamine (TAMRA) as acceptor on each end. B) Normalized fluorescence spectrum of duplex RNA (13 bp) incubated with (green line) or without (red line) RNase/serum at 25°C for 15 min. C) Duplex RNA degradation assay measurement in real-time. Duplex RNA was annealed and incubated in annealing buffer at 25°C. RNase or serum was added at the time indicated. Fluorescence of the donor (FAM) was recorded in real-time (solid dots). Samples at different time (from left to right: 0 s, 10 s, 60 s, 180 s, 480 s) were collected and run in a denatured PAGE gel (Inset). Fluorescence was scanned on Typhoon showing the remaining amount of full length dsRNA.

Different from single-stranded RNAs, double-stranded RNAs are generally much more stable. In a recent study profiling the serum degradation of double-stranded siRNAs, we found that both long and short double-stranded RNAs are degraded predominately at two susceptible sites, namely 5′-U/A-3′ and 5′-C/A-3′ dinucleotide sites [31]. Based on this finding, we speculated that double-stranded RNAs might be used as a substrate for measuring serum RNase activity. To this end, a double-stranded RNA sequence was designed to carry a single 5′-C/A-3′ susceptible site, which was confirmed in degradation assay (Figure 1A). RNase A is the predominant RNase in human serum that mediates the degradation process of such dsRNAs (Figure 1B). To facilitate the monitoring of the degradation process of this probe in real time, a FRET system was designed by integrating a donor fluorescence FAM and an acceptor fluorescence TAMRA at the 5′ end of each component RNA strands. As long as the RNA duplex keeps intact, the two end-labeled fluorescent dyes are close enough to mediate energy transfer from the donor to the acceptor (Figure 2A), leading to an emission peak at 575 nm when excited at 480 nm (Figure 2B). When the duplex is degraded, the energy transfer from FAM to TAMRA will be interrupted, and emission peak will shift from 575 nm to 515 nm, under the same excitation conditions (Figure 2B). Therefore, measuring the change of the emission peak at 515 nm can be used to monitor the degradation process of the duplex RNA and reflect the activity of the RNase in the system (Figure 2C).

It is well established that the energy transfer efficacy depends mainly on the distance and the relative orientation of the donor and the acceptor fluorescence in the probe [32]. On one hand, a short probe increases the energy transfer efficiency in the FRET, while on the other hand, a longer probe has a higher melting temperature and is more stable. RNA duplexes of 8 and 13 bp were studied to optimize the length of the substrate. The results showed that the 13-bp substrate, with sequences 5′-AUGAGCCUGAUUU-3′/3′-UACUCGGACUAAA-5′, was optimal for FRET detection. This RNA duplex was then used as the reaction substrate in the study.

### Optimization of the Assay System

**Figure 3:**
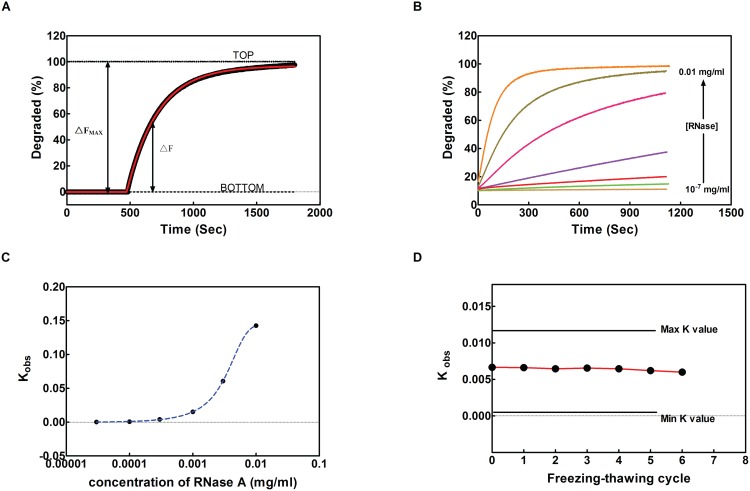
FRET assay method validation. A) Quantification of degradation in FRET assay. The FAM fluorescent intensity is converted as degradation ratio by defining bottom and top fluorescent intensity as 0% and 100%. Bottom is the fluorescent intensity of full length dual-labeled dsRNA; top is the fluorescent intensity of FAM labeled single strand RNA. The ΔF_max_ was defined as 100%. The Fluorescent readout of samples was fitted to the single-exponential equation (red line) to obtain the rate constant K_obs_. Percent degradation at a given time was calculated as 100×ΔF/ΔF_max_. B) Degradation of dual-labeled dsRNA at various concentration of RNase A, monitored by FRET assay (from bottom to top: 10^−7 ^mg/ml, 10^−6 ^mg/ml, 10^−5 ^mg/ml, 0.001 mg/ml, 0.003 mg/ml, 0.006 mg/ml, 0.01 mg/ml). C) Standard curve of RNase A concentration and K_obs_. K_obs_ were obtained as described in Figure 3A. D) K_obs_ changing pattern under freezing-thawing treatment. K_obs_ is determined from human serum samples frozen in −80°C, and thawed in room temperature from 0 to 6 times.

To measure RNase activity in the system, four microliters of 5 µM dual-labeled RNA substrate were added into 2 ml FRET buffer under constant stirring. Before adding of RNase or test samples, a baseline curve was recorded at 480 nm excitation for about 30 seconds, using a QuantaMaster 30 spectrofluorometer (Photon Technology International, Birmingham). Then RNase solution or test samples were added to the reaction system, and the fluorescence was recorded at 515 nm at an interval of 3-second for 15 minutes. For data analysis, the background fluorescence was subtracted from the sample fluorescence to obtain a real-time curve, so as to monitor the degradation process in real-time during the treatment (Figure 2C). A nonlinear regression method (single-exponential equation) was used to fit the data and obtain the constant K_obs_ of the degradation reaction (Figure 3A).

In such a way, a standard curve was established by measuring RNase A activity in serially diluted RNase A solutions, in a concentration range between10^−7^ to 10^−1^ µg/µl (Figure 3C). For each concentration, a kinetic curve was recorded (Figure 3A), and a reference curve was made according to the calculated K_obs_ to describe the relationship between the activity and the concentration of RNase A. A simulated curve showed that the degradation activity rose rapidly when the RNase concentration was increased (Figure 3C). The results showed that a linear relationship over a wide range of RNase A concentrations from 10^−7^ to 10^−3^ µg/µl. After adding RNase A 10^−7^ µg/µl, fluorescent signal intensity increased 1000 a.u., the signal noise ratio was 2 fold. The detection limit of FRET assay was 10^−7^ µg/µl. When the RNase assay was performed with 1×10^−6^ µg/µl RNase A, the fluorescent signal intensity increased from 41000 a.u. to 58000 a.u., and the noise in this assay was 500. The signal noise ratio under 1×10^−6^ µg/µl is 34 fold. According to this signal noise ratio, the detectable range was as low as1×10^−6^ µg/µl. The nonlinear regression R^2^ at 1×10^−6^ µg/µl concentration was 0.9856. Under the concentration of 1×10^−5^ µg/µl, the nonlinear regression R^2^ was 0.9945. On the other hand, the fluorescent signal intensity increased from 43000 a.u. to 78000 a.u., and the signal noise ratio was 70 fold. According to the R^2^ and the signal noise ratio, we concluded that the concentration range of RNases that can be readily measured by this method should be from 1×10^−5^ to 0.1 µg/µl.

To further validate this assay system, RNase activities were measured for human serum samples that have undergone repeatedly freezing-thawing treatment. By freezing the samples at −80°C and thawing on ice for multiple times, the RNase activities of the samples were measured using the current FRET method. It was found that RNase could survive multiple freezing-thawing cycles without any loss of activity, indicating the current method can be used for frozen clinical samples (Figure 3D).

### Measuring Serum RNase Activity in Cancer Patients

**Figure 4:**
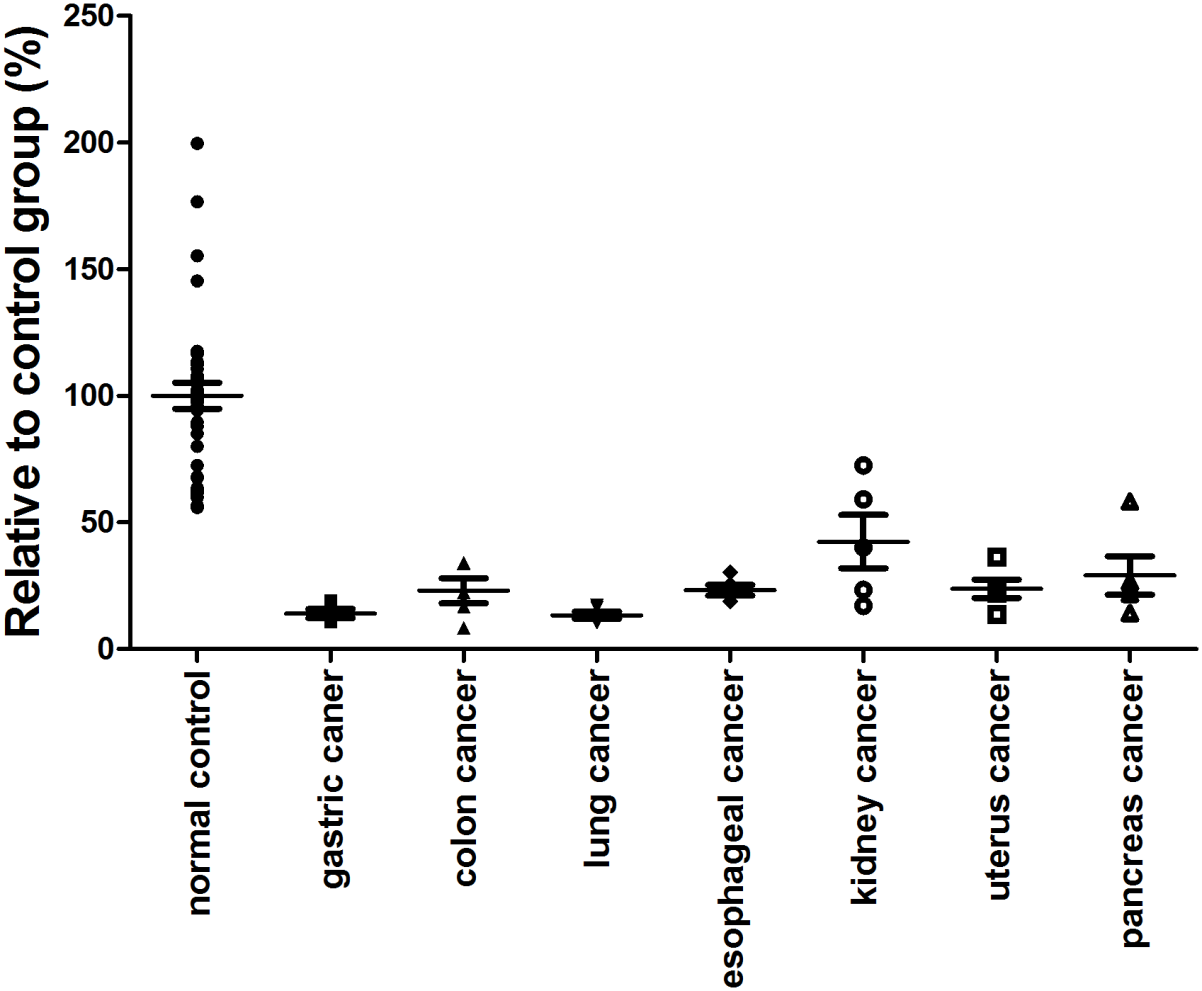
General down-regulation of serum RNase levels in cancer patients. Serum RNase activities were determined for healthy individuals as well as 4 gastric cancer patients, 5 colon cancer patients, 5 lung cancer patients, 5 esophageal cancer patients, 5 kidney cancer patients, 5 uterus cancer patients and 5 pancreatic cancer patients. The RNase concentration of each serum sample was calculated by the standard curve and K_obs_ obtained from single-exponential equation. The relative serum RNase activity of each cancer patient was quantified by normalizing the cancer patient serum concentration with the average concentration of healthy individuals’ serum samples.

Using this FRET-based measurement of RNase activity, a set of human serum samples were studied, including 55 healthy individuals and 34 cancer patients covering some common cancer types. The standard curve described above was used to calculate the relative serum RNase concentrations of human serum samples, and then the relative RNase concentration of cancer patients were compared to that of the healthy individuals (average of normal controls arbitrarily was set to 100%). The results showed that serum RNase levels were significantly down-regulated for all 7 kinds of cancer types (Figure 4).

**Figure 5:**
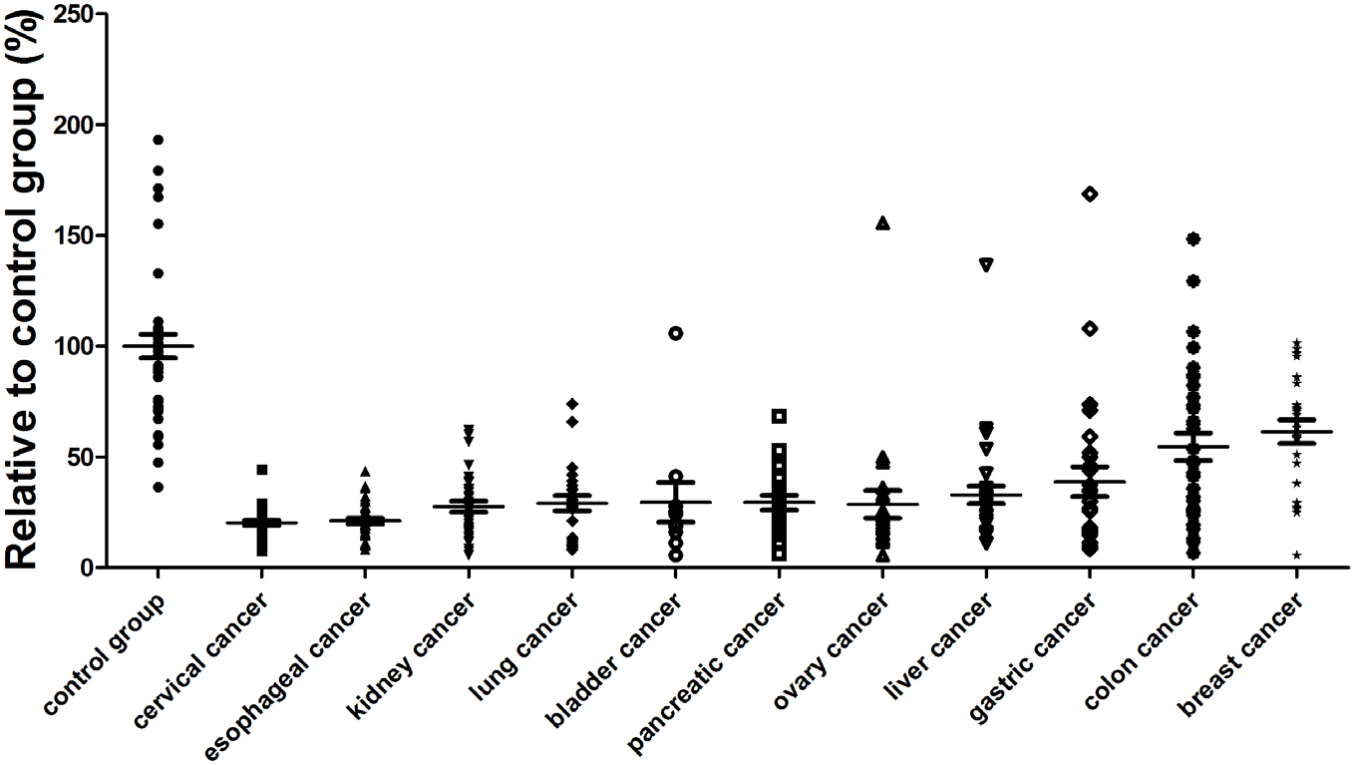
Distinct RNase activities of healthy individuals and cancer patients. Serum RNase levels were quantified for healthy individuals as well as 37 cervical cancer patients, 37 esophageal cancer patients, 37 kidney cancer patients, 24 lung cancer patients, 10 bladder cancer patients, 21 pancreatic cancer patients, 23 ovary cancer patients, 32 liver cancer patients, 27 gastric cancer patients, 31 colon cancer patients, and 24 breast cancer patients (for more details of data see Table S1).

To confirm these observations, additional serum samples were collected from patients with breast cancer, gastric cancer, colon cancer, liver cancer, pancreatic cancer, esophageal cancer, ovary cancer, cervical cancer, bladder cancer, kidney cancer and lung cancer, at a sample size of 303 cancer patients in total. Assessment of these samples demonstrated a down-regulation of serum RNase levels in gastric cancer, liver cancer, pancreatic cancer, esophageal cancer, ovary cancer, cervical cancer, bladder cancer, kidney cancer and lung cancer (Table S1). No evident changes of serum RNase levels were observed for breast cancer patients and colon cancer patients. These results largely supported our earlier observations. The gastric cancer, liver cancer, pancreatic cancer, esophageal cancer, ovary cancer, cervical cancer, bladder cancer, kidney cancer and lung cancer patient groups differed from healthy group with a P value less than 0.001. In comparison, the P value for breast cancer group was only 0.0619, showing no obvious difference from the control group (Figure 5). Taken together, these data indicated that down-regulation of serum RNase activity is a general phenomenon in many common cancer types. This observation hinted a potential for site-specific RNase as a common cancer biomarker.

There were 32 liver cancer tested, the average relative activity compared with normal control was 32.9%, with P<0.0001. Out of the 32 samples, only 1 sample fell into control activity range. Twenty seven gastric cancer patients’ serum were tested, the mean relative activity was 38.8%, with P<0.001. There were 24 lung cancer patients’ serum were tested, the average relative activity was 29.1%, P<0.0001. Thirty seven esophageal cancer patients’ serum and 35 cervical cancer patients’ serum, 37 kidney cancer patients’ serum and 21 pancreatic cancer were tested and shown to have an 21.1% with P<0.0001, 20.2% with P<0.0001, 27.6% with P<0.0001, 29.4% with P<0.0001, respectively. There were 23 ovary cancer patients’ sera were measured, the relative activity of ovary cancer was 28.6%, with P<0.0001, and one case was fallen into the control range. For bladder cancer we also observed an exceptional case that fell into the normal range while the average relative activity was 29.5%, with P<0.0001. The breast cancer did not share the similar down-regulated RNase pattern with all the other 10 kinds of cancers, the relative activity of breast cancer was 61.4%, slightly lower than the normal individuals, but statistical analysis suggested such a difference might not be significant (P = 0.0619). A similar situation was encountered with colon cancer patients studied with the average relative RNase A activity at 54.5% and P<0.05.

### No Difference in Serum RNase Levels between Patients with Primary and Metastatic Colon Cancer

**Figure 6:**
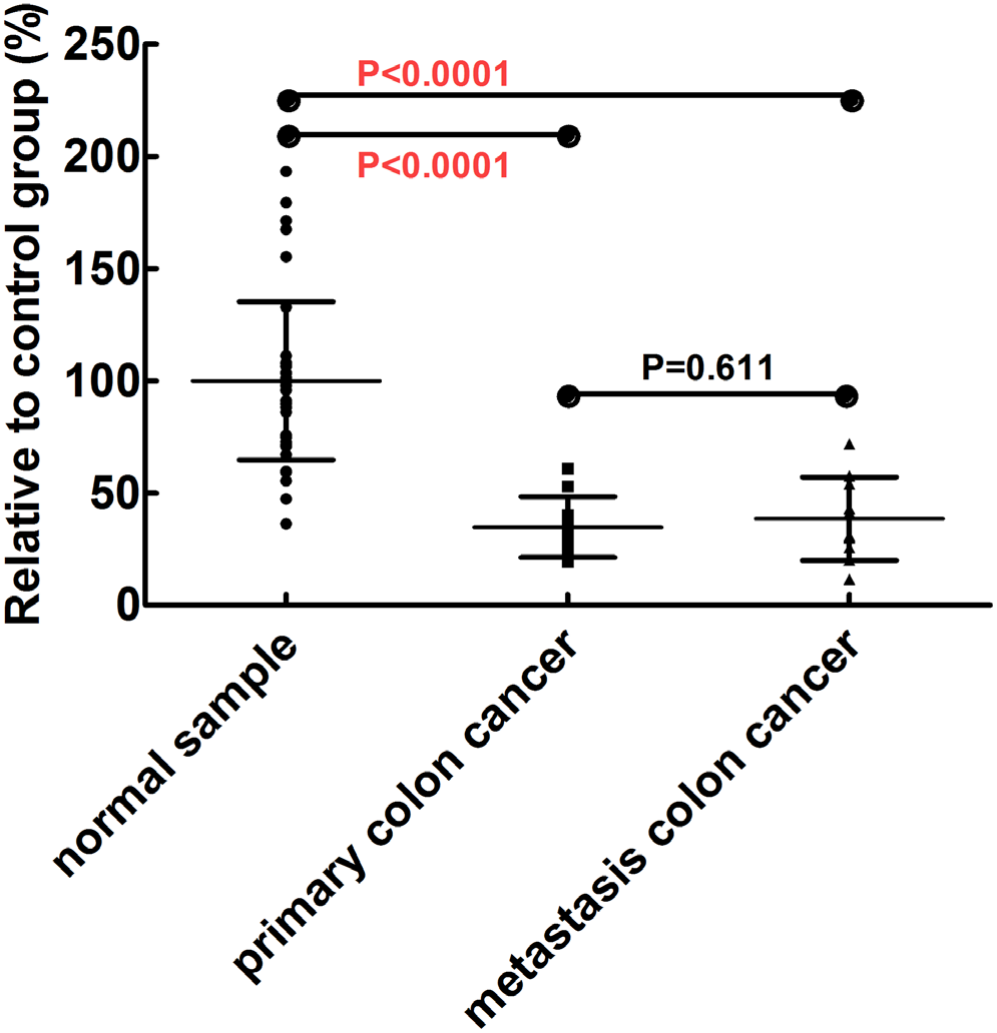
Serum RNase activities of patients with primary and metastatic colon cancer. These rum RNase activities of 10 primary colon cancer patients and 10 metastasis colon cancer patients were quantified by FRET assay.

RNase levels were further examined in primary and metastatic colon cancer patients, in order to explore if the wide-spread values of RNase activities in serum of colon cancer patients were due to mixing of stages, and if RNase activity differences can be used to discriminate different stages of this cancer. Serum samples were collected from primary and metastasis colon cancer patients for whom the metastasis stages were determined by examination of tissue biopsy. Serum samples from ten patients with lymph-node metastases and 10 without metastases were collected and analyzed, using the FRET RNase assay. The results showed no difference between the two groups (Figure 6).

## Discussion

A correlation between serum RNase levels and pancreatic cancer was first reported by Reddi in 1976. Using an optical method, they measured the level of serum RNase by quantifying its activity on poly-C RNA, a non-specific RNase substrate [19]. In late 1970s and early 1980s, radioimmunoassay was implemented in RNase measurement [24], [25], and in some studies E. coli tRNA was used to replace poly-C as assay substrate [26], these improvements enhance the measurement accuracy of RNase. Several studies have been carried out to investigate the serum RNase levels in patients with malignant carcinoma, benign tumor, smoker, renal failure, and in particularly patients with pancreatitis and pancreatic cancer. These studies however revealed a mixed picture [22], [24]. Although Funakushi and Kemmer groups reported increased serum RNase levels in patients with both pancreatic cancer and pancreatitis [21], [26], Reddi and Warshaw groups however found that the RNase level increased only in pancreatic cancer patients [19], [22], whereas the serum RNase levels of pancreatitis patients were at a similar level as healthy controls [19], [22]. Mitsuhashi and Kurihara groups, on the other hand, found that the serum RNase levels related only with the age, smoking as well as some other physiological index, including blood urea nitrogen and albumin contents [25]. In addition, a recent study reported elevated serum RNase levels in patients with juvenile diabetes mellitus [33]. Yet another report shown that RNase 1 has changed its expression level by microarray and western blotting method during the gastric cancer development [34].

These discrepancies may be caused by the complexity of Reddi’s assay, for example the reaction termination step or the substance purification steps. In these assays, the reaction systems were let on ice for quite a while, before high dose of HClO_4_ was added to stop the digestion. However in our study, it was noted that ice bath treatment could not completely abolish RNase activity. Additional, purification of undigested polynucleotide might be another step of complication for Reddi’s assay. In this step a simple centrifugation was used to remove di-nucleotide and tri-nucleotide resulted from RNase digestion. This process however also might also remove some longer polynucleotides that were not completely digested, therefore leads to over-estimated activity of serum RNase.

A FRET assay was used in our study to record the fluorescent intensity in real time, so as to avoid the reaction termination process and the purification step. Recording the fluorescence signal changes in real time, and using a FRET probe that has only one RNase A cleavage site, apparently enabled an accurate calculation of the K_obs_ for the reaction.

In the present study, a FRET-based method was established to measure serum RNase levels consistently with a high sensitivity to detect as low as 1×10^−7^ µg/µl RNase. In this study, the performance of spectrofluorometer was systematically optimized using Raman spectrum of water. This was found to enhance the quality of the measurement and reproducibility between tests. By applying a 2 ml reaction volume, we were actually able to measure serum RNase level without serial dilution, thus avoided potential deviation caused by a 1000 time dilution in other protocols.

The re-discovery of the RNA world has drawn more and more attention to a variety of RNases functioning in RNA modification and metabolism. Thus a reliable method of RNases A detection can be plausible for such studies. The FRET assay established in the present study is not only limited to RNase measurement in serum samples, but can also be used in many other clinical tests and scientific analyses. With interpretation of the results, such as observed decrease of RNases A activity in serum of certain type of cancer patients, a grain of caution need to be taken, as we were not taking medication of different patients into account, and there is a need to determine whether any medication can inhibit the RNase A activity. Staging of patients subjected to serum RNase measurement is obviously one of the most important factors to consider before enlisting RNase as a potential biomarker, and for this reason more patients with early stage cancers should be tested to trace the initial detection of decrease of RNase A activity.

## Materials and Methods

### Human Sample Collection and Ethics Statement

This study has followed the Declaration of Helsinki, and conducted according to the principles approved by the Ethics Review Boards of the Cancer hospital, Chinese Academy of Medical Sciences (Beijing, China). Written informed consent was provided for sample collection and subsequent analysis. In total, 431 serum samples were collected from healthy individuals and cancer patients and analyzed in the study. The characteristics of cancer patient were described in Table 1. The sera were stored at −80°C after collection.

**Table 1:** Characteristics of the studied cancer patients (n = 303).

Characteristics	n (%)
**Age (yr)**	
range	20–82
mean±SD	54.6±11.9
Median	55
**Gender**	
Male	157 (52)
Female	146 (48)
**Age distribution**	
20–29	4 (1.3)
30–39	24 (7.9)
40–49	84 (27.7)
50–59	86 (28.4)
60–69	68 (22.4)
70–79	33 (10.9)
80–89	4 (1.3)

### Oligonucleotide Synthesis and Preparation

Two complementary and fluorophore-labeled RNA oligonucleotides with sequences of 5′-FAM-AUGAGCCUGAUUU and 5′-TAMRA-AAAUCAGGCUCAU were synthesized and purified by TAKARA (Dalian, China). Concentrations of the RNA oligonucleotide were determined by measuring the absorption at 260 nm, using a Nanodrop spectrophotometer. The extinction coefficient was also measured at 260 nm, resulting in 140860 L/(mol*cm) for the FAM-labeled RNA oligonucleotide and 156900 L/(mol*cm) for the TAMRA-labeled RNA oligonucleotide. The two complementary RNA oligonucleotides were mixed in an annealing buffer (5 mM Tris-HCl, pH = 7.6, 10 mM NaCl) at a concentration of 5 µM. The mixture was incubated at 95°C for 5 min in a thermal cycler, and then the temperature was decreased by 5°C for every 5 min to allow duplex formation. The resulting duplexes were checked in a 20% polyacrylamide gel and visualized by SYBR Gold staining (Molecular Probes, Eugene, OR, USA).

### FRET Assay

Fluorescence measurements were carried out in FRET buffer (0.01 M Tris-HCl, pH 7.4, 0.002 MgCl_2_), using a QuantaMaster 30 spectrofluorometer (Photon Technology International, Birmingham).

In 2 ml FRET buffer, 10 nM RNA duplex was added under constant stirring. The excitation wavelength was set at 480 nm, and the emission spectrum signal was measured between 500 to 650 nm. An increase in the fluorescence emission at 515 nm indicates the progress of RNA cleavage. An example of data collected is presented in Figure 2C.

For data normalization, an intact 13 bp siRNA was analyzed as a duplex control, and a completely cleaved siRNA was included as a degradation control. The FAM fluorescent intensities were converted as degradation ratio by defining bottom and top fluorescent intensities as 0% and 100%. The normalization method was mentioned in Figure 3. The Fluorescent readings were fitted to the single-exponential equation (red line) to obtain the rate constant K_obs_. Percent degradation at a given time was calculated as 100×ΔF/ΔF_max_ (Figure 3A).

To ensure the RNase-free condition, we set the excitation wavelength at 480 nm, and examined the emission between 500 to 650 nm of intact substrates. Intact substrate shows low signal at 515 nm and high signal at 575 nm, while degraded products results in elevated signals at 515 nm.

According to the description above, when the dual labeled 13-bp siRNA in 2 ml FRET buffer mixed with RNase A solution or serum samples, the fluorescence of FAM would be increased. The change of fluorescence was monitored over time with excitation wavelength set at 480 nm and emission at 515 nm. For single-turnover kinetics analysis, the rate constant k_obs_ and amplitude of maximal degradation F_max_ was derived by fitting the experimental data to the single-exponential equation:
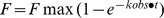
_._

### Identification of RNase Cleavage Sites

To pinpoint RNase cleavage sites, dsRNA degradation products were extracted and cloned using as mall RNA gel extraction kit and cloning kit from TaKaRa (Kyoto, Japan). In detail, the degradation products were dephosphorylated by alkaline phosphatase and then ligated to a biotin-labeled RNA/DNA hybrid 3′adaptor. After purification by means of streptavidin bead binding, the fragments were phosphorylated and ligated to another RNA/DNA hybrid 5′adaptor. The resulting products (5′adaptor-RNA fragment-3′adaptor) were reverse-transcribed, amplified by PCR, cloned into T vector, and finally sequenced. Sequences of the cleavage products were aligned back onto the dsRNA directionally to deduce the RNase cleavage sites [31].

## Supporting Information

Table S1 Serum RNase activities of 11 kinds of cancer patients.
(DOCX)
