# Supplementary material for: Site-Specific RNase A Activity Was Dramatically Reduced in Serum from Multiple Types of Cancer Patients
Source: PLoS One. 2014 May 7;9(5):e96490. doi: 10.1371/journal.pone.0096490 (PMC4013009; doi:10.1371/journal.pone.0096490)
Supplement: Table S1 — Serum RNase activities of 11 kinds of cancer patients. (DOCX) [file pone.0096490.s001.docx]

Supplementary table S1

| **Cancer type** | **Relative average of RNase A concentration (%)** | **Standard Deviation (%)** | **Nmber of patients** | **P value(compared with control individuals)** |
| --- | --- | --- | --- | --- |
| Cervical cancer | 20.19 | 7.4081 | 35 | <0.0001 |
| Esophageal cancer | 21.08 | 8.0435 | 37 | <0.0001 |
| Kidney cancer | 27.57 | 14.4957 | 37 | <0.0001 |
| Lung cancer | 29.10 | 16.4828 | 24 | <0.0001 |
| Bladder cancer | 29.46 | 27.079 | 10 | <0.0001 |
| Pancreatic cancer | 29.39 | 15.0954 | 21 | <0.0001 |
| Ovary cancer | 28.60 | 29.0759 | 23 | <0.0001 |
| Liver cancer | 32.85 | 22.2728 | 32 | <0.0001 |
| Gastric cancer | 38.78 | 34.3946 | 27 | <0.001 |
| Colon cancer | 54.50 | 26.8336 | 33 | 0.034 |
| Breast cancer | 61.39 | 25.624 | 24 | 0.0619 |
